# Elevated Expression of Vacuolar Nickel Transporter Gene *IREG2* Is Associated With Reduced Root-to-Shoot Nickel Translocation in *Noccaea japonica*

**DOI:** 10.3389/fpls.2020.00610

**Published:** 2020-06-03

**Authors:** Sho Nishida, Ryoji Tanikawa, Shota Ishida, Junko Yoshida, Takafumi Mizuno, Hiromi Nakanishi, Naoki Furuta

**Affiliations:** ^1^Laboratory of Plant Nutrition, Faculty of Agriculture, Saga University, Saga, Japan; ^2^Laboratory of Environmental Chemistry, Faculty of Science and Engineering, Chuo University, Tokyo, Japan; ^3^Laboratory of Soil Science and Plant Nutrition, Graduate School of Bioresources, Mie University, Tsu, Japan; ^4^Laboratory of Plant Biotechnology, Graduate School of Agricultural and Life Sciences, The University of Tokyo, Tokyo, Japan

**Keywords:** iron regulated 2, metal hyperaccumulator, nickel, nicotianamine, *Noccaea*, phytoremediation, transporter, zinc

## Abstract

A number of metal hyperaccumulator plants, including nickel (Ni) hyperaccumulators, have been identified in the genus *Noccaea*. The ability to accumulate Ni in shoots varies widely among species and ecotypes in this genus; however, little is known about the molecular mechanisms underlying this intra- and inter-specific variation. Here, in hydroponic culture, we compared Ni accumulation patterns between *Noccaea japonica*, which originated in Ni-enriched serpentine soils in Mt. Yubari (Hokkaido, Japan), and *Noccaea caerulescens* ecotype Ganges, which originated in zinc/lead-mine soils in Southern France. Both *Noccaea* species showed extremely high Ni tolerance compared with that of the non-accumulator *Arabidopsis thaliana*. But, following treatment with 200 μM Ni, *N. caerulescens* showed leaf chlorosis, whereas *N. japonica* did not show any stress symptoms. Shoot Ni concentration was higher in *N. caerulescens* than in *N. japonica*; this difference was due to higher efficiency of root-to-shoot Ni translocation in *N. caerulescens* than *N. japonica*. It is known that the vacuole Ni transporter IREG2 suppresses Ni translocation from roots to shoots by sequestering Ni in the root vacuoles. The expression level of the *IREG2* gene in the roots of *N. japonica* was 10-fold that in the roots of *N. caerulescens*. Moreover, the copy number of *IREG2* per genome was higher in *N. japonica* than in *N. caerulescens*, suggesting that *IREG2* expression is elevated by gene multiplication in *N. japonica*. The heterologous expression of *IREG2* of *N. japonica* and *N. caerulescens* in yeast and *A. thaliana* confirmed that both *IREG2* genes encode functional vacuole Ni transporters. Taking these results together, we hypothesize that the elevation of *IREG2* expression by gene multiplication causes the lower root-to-shoot Ni translocation in *N. japonica*.

## Introduction

In plants, heavy metals absorbed from the soils by the roots accumulate in roots and shoots. Some heavy metals, such as zinc (Zn), manganese (Mn), and copper (Cu) are essential nutrients for plants, but excess accumulation of these metals can be toxic. Thus, plants found in natural environments contain heavy metals within certain ranges of concentrations, which can be defined as the “normal” ranges (Marschner, 1995). Some plant species, however, accumulate heavy metals in their aerial parts at unusually high levels without any stress symptoms. These species are called “metal hyperaccumulators” (Brooks et al., 1977). The threshold that defines metal hyperaccumulation depends on the element; for example, Zn/Mn hyperaccumulators contain >10,000 mg kg^–1^, and nickel (Ni)/Cu hyperaccumulators contain >1,000 mg kg^–1^ on a dry weight basis (Reeves et al., 2018). More than 700 species have been identified as metal hyperaccumulators, and most of these have been discovered in metal-contaminated soils (Reeves et al., 2018). Metal hyperaccumulators have been studied both as rare species with unique molecular mechanisms for abnormal metal accumulation, and as model organisms for adaptation to extreme environments (Hanikenne and Nouet, 2011). Metal hyperaccumulators have also received attention from researchers developing technologies for decontaminating metal-polluted soils using plants, so-called “phytoremediation,” or for harvesting rare metals from soils, so-called “phytomining” (Salt et al., 1998; Chaney et al., 2007).

Metal hyperaccumulators have several characteristic traits: enhanced metal absorption by roots, efficient metal translocation from roots to shoots, and elevated metal tolerance (Lasat et al., 1996; Kramer et al., 1997; Zhao et al., 2002; Caille et al., 2005). In recent years, the molecular mechanisms involved in those traits have been uncovered by molecular biological approaches. Zn/cadmium (Cd) transporter Heavy Metal ATPase 4 (HMA4), which plays a role in Zn/Cd loading into the xylem in roots, was shown to be involved in efficient Zn/Cd translocation from roots to shoots in the Zn/Cd hyperaccumulator *Arabidopsis halleri* using *HMA4*-knockdown plants (Hanikenne et al., 2008). HMA3 is thought to play a role in sequestering excess Cd in leaf vacuoles and contribute to high Cd tolerance and accumulation in shoots of the Zn/Cd/Ni hyperaccumulator *Noccaea caerulescens* (formerly *Thlaspi caerulescens*) ecotype Ganges (Ueno et al., 2011). Natural resistance-associated macrophage protein 1 (NRAMP1) is thought to mediate Cd influx across the endodermal plasma membrane in root vascular tissues and contribute to efficient root-to-shoot Cd translocation in *N. caerulescens* ecotype Ganges (Milner et al., 2014). The expression levels of *HMA4*, *HMA3* and *NRAMP1* are much higher in the metal hyperaccumulators than in the non-accumulators, and the elevated expression levels are required for metal hyperaccumulating abilities (Hammond et al., 2006; Hanikenne et al., 2008; Ueno et al., 2011; Milner et al., 2014). Hanikenne et al. (2008) attributed the elevated expression of *HMA4* in *A. halleri* to the triplication of *HMA4* in the genome and the mutation of *cis*-regulatory sequences. Elevated expression of *HMA4* caused by gene multiplication is also found in the Zn/Cd hyperaccumulating ecotypes of *N. caerulescens* (Lochlainn et al., 2011). Elevated *HMA3* and *NRAMP1* expression levels in *N. caerulescens* ecotype Ganges are also likely caused by gene multiplication (Ueno et al., 2011; Milner et al., 2014), indicating that the elevated expression of metal transporter genes caused by gene multiplication are key evolutionary events for metal hyperaccumulators. High expression of many other genes involved in metal transport and metal homeostasis is also thought to be implicated in metal hyperaccumulation (Hanikenne and Nouet, 2011).

Most metal hyperaccumulators (more than 500 species) are Ni hyperaccumulators (Reeves et al., 2018). Ni hyperaccumulators are found on serpentine soils, which contain Ni at a level excessive for normal plants. In the Ni hyperaccumulating ecotype of *N. caerulescens*, nicotianamine in root cells and xylem forms a complex with Ni and is involved in the long-distance translocation of Ni from roots to shoots (Vacchina et al., 2003; Mari et al., 2006). Expression of *Yellow-stripe like 3* (*YSL3*), encoding the Ni–nicotianamine complex transporter, is higher in the roots and shoots of *N. caerulescens* than in those of the non-accumulator *Arabidopsis thaliana* (Gendre et al., 2007), suggesting that elevated *YSL3* expression increases Ni–nicotianamine mobility and contributes to efficient root-to-shoot Ni translocation. In another *Noccaea* Ni hyperaccumulator, *N. goesingense*, elevated serine acetyltransferase activity and elevated glutathione concentration are essential for enhanced Ni tolerance (Freeman et al., 2004, 2005; Na and Salt, 2011).

Assunção et al. (2003) showed that accumulation patterns of Ni, Zn, and Cd vary among *N. caerulescens* ecotypes originating from different soil types, and that an ecotype growing in serpentine soils is superior in Ni tolerance and accumulation to ecotypes from other metalliferous or non-metalliferous soils. Peer et al. (2003, 2006) showed that there is a large variation in Ni accumulation patterns among the species or accessions of *Brassicaceae* metal hyperaccumulators including a number of *Noccaea* species. In their results, shoot Ni concentration was not always correlated with the shoot concentrations of other metals, suggesting that there is a genetic factor specifically determining Ni hyperaccumulation.

The molecular mechanism of Ni accumulation has been well studied in *A. thaliana*. Ni is absorbed by roots via iron (Fe) uptake transporter Iron-regulated transporter 1 (IRT1) and unknown transporters (Nishida et al., 2011, 2015), and then translocated to shoots via the xylem. Excessively absorbed Ni is sequestered in root vacuoles by Ni transporter Iron-regulated 2 (IREG2) (also known as Ferroportin 2; Schaaf et al., 2006; Morrissey et al., 2009). Excess Ni induces Fe deficiency due to competition between Ni and Fe (Nishida et al., 2012). Then, *IRT1* expression is induced by the Ni-induced Fe deficiency, resulting in accelerated Ni absorption via IRT1 (Nishida et al., 2012). *IREG2* is also induced by Ni-induced Fe deficiency and detoxifies excess Ni (Schaaf et al., 2006). This synchronized induction of *IREG2* and *IRT1*, which is regulated by FER-like Fe deficiency-induced transcription factor (FIT), is essential for the limited Ni tolerance shown by *A. thaliana* (Schaaf et al., 2006). Knockdown of *IREG2* decreases root Ni and increases shoot Ni, indicating that *IREG2* can determine the efficiency of Ni translocation from roots to shoots (Schaaf et al., 2006; Merlot et al., 2014). IREG1, the ortholog of IREG2, plays a role in Fe/cobalt (Co) translocation from roots to shoots by loading Fe/Co into the xylem in roots, but the contribution of IREG1 to Ni translocation was not confirmed (Morrissey et al., 2009). Merlot et al. (2014) reported that the expression level of a *IREG* homolog is higher in the Ni hyperaccumulator *Psychotria gabriellae* than in the related non-accumulator *Psychotria semperflorens* and the overexpression of *P. gabriellae IREG* increases Ni tolerance in yeast and *A. thaliana*, suggesting that *IREG* contributes to high Ni tolerance in *P. gabriellae*.

The objective of the present study was to reveal a genetic factor associated with variation in shoot Ni accumulation in Ni hyperaccumulators. We assessed Ni accumulation patterns in roots and shoots in two related Ni hyperaccumulators: *Noccaea japonica*, which originated from a serpentine soil, and *N. caerulescens* ecotype Ganges, which originated from a Zn/Pb-mine soil. We also investigated the involvement of *IREG2* in the differential Ni accumulation patterns between the two *Noccaea* plant types.

## Materials and Methods

### Plant Materials

*Noccaea japonica* growing in a serpentine soil in Mt. Yubari (Hokkaido, Japan), *N. caerulescens* ecotype Ganges growing in a Zn/Pb-mine soil in Southern France, and *A. thaliana* accession Col-0 were used. The *ireg2-1* T-DNA insertion line (SALK_74442; Schaaf et al., 2006) was obtained from the Arabidopsis Biological Resource Center (Ohio State University, United States). An *ireg2-1* homozygous line was identified using polymerase chain reaction (PCR) with primers flanking the insertion site and the left-border primer of T-DNA (Supplementary Table S1).

### Growth Conditions

Half-strength Molecular Genetics Research Laboratory (MGRL) medium containing 8.6 μM Fe-EDTA at pH 5.5 was used as the hydroponic culture solution (Nishida et al., 2011). The procedure used for hydroponic culture was described previously (Nishida et al., 2011). Since *N. japonica* and *N. caerulescens* grow slower than *A. thaliana*, the preculture periods prior to metal exposure were 6 weeks for the *Noccaea* species and 4 weeks for *A. thaliana*. Two plants were cultured in every pot and pooled as one biological replicate. Plants were grown in a growth chamber at 22°C under a 10-h light/14-h dark cycle.

In agar-plate tests, half-strength MGRL medium containing 1.2% agar (agar type A, Sigma-Aldrich Co), 1% sucrose, 8.6 μM Fe-EDTA, and 5 mM 2-morpholinoethanesulfonic acid (MES) at pH 5.5 was used. Seeds were surface sterilized and sown on agar plates. Fifteen seedlings were pooled as one biological replicate. Plants were cultured in a growth chamber at 22°C under a 16-h light/8-h dark cycle.

### Determination of Metal Tolerance and Accumulation in Hydroponic Culture

Plants were exposed to hydroponic culture solutions supplemented with 0–200 NiCl_2_ or 0.5–1000 μM ZnCl_2_ for a week. Hydroponic solutions were buffered with 5 mM MES (pH 5.5). After exposure, plants were separated into roots and shoots. Roots were washed with deionized water and 50 mM EDTA-Na_2_ to remove metals bound to the cell walls, and then blotted. Harvested samples were weighed to determine the fresh weight, dried at 70°C, and weighted to determine the dry weight. Dried samples were digested with HNO_3_ and H_2_O_2_ in a heat block and diluted with ultra-pure water. Elemental analysis was performed using inductively coupled plasma optical emission spectrometers (ICPS-7500, Shimadzu Co.; SPS5100, Hitachi High-Tech Science Co).

### Cloning of *IREG2* From *Noccaea* Species

*Noccaea japonica* and *N. caerulescens* plants were cultured hydroponically as described above. Total RNA was extracted from their roots following the method of Suzuki et al. (2004). Residual DNA was digested by DNase I (Takara Bio Inc), and first-strand cDNA was synthesized using a PrimeScript RT-PCR kit (Takara Bio Inc) with oligo (dT) primers. The resulting cDNA was used for gene cloning as follows. The mRNA sequence of *NcIREG2*, which was previously obtained by the Transcriptome Shotgun Assembly project for *N. caerulescens*, is available at DDBJ/EMBL/GenBank as “Nc_isotig06993_isogroup03561” (Lin et al., 2014; Supplementary Figure S3). Full-length *IREG2* fragments from *N. japonica* and *N. caerulescens* were amplified using forward and reverse primers designed on regions corresponding to the 5’ UTR and 3’ UTR of *NcIREG2*, respectively, and a high-fidelity DNA polymerase (PrimeSTAR HS DNA Polymerase, Takara Bio Inc). PCR products were sequenced using primers designed on the internal regions of *IREG2* (Supplementary Table S1). Since single nucleotide polymorphisms were detected in *NjIREG2*, *NjIREG2* fragments were cloned separately in an *Escherichia coli* plasmid vector and sequenced as described later. *IREG2* sequences were aligned by using Clustal W^1^. *IREG2* sequences are deposited in the DNA Data Bank of Japan under the accession numbers LC522035 (*NcIREG2*) and LC522036 (*NjIREG2*).

### Quantitative Reverse Transcription (RT)-PCR Assay

Plants pre-cultured in hydroponic solution were exposed to 200 μM NiCl_2_ for a week. Total RNA was then extracted from the roots and treated with DNase I. cDNA was synthesized using a ReverTra Ace qPCR RT kit (Toyobo Co., Ltd), and quantitative RT-PCR (qRT-PCR) was performed using THUNDERBIRD SYBR qPCR Mix (Toyobo Co., Ltd). qPCR was performed in a real-time PCR system (LightCycler 96 System, Roche Diagnostics KK). The relative transcript level of *IREG2* was calculated using a calibration curve and normalized with the *EF1*α transcript level. The *IREG2*-specific primers were designed on sequences that are completely identical between *NcIREG2* and *NjIREG2*. To design *EF1*α-specific primers, a portion of the *EF1*α sequence was amplified from genomic DNA of *N. japonica* and *N. caerulescens* by PCR with *EF1*α common primers, which were designed on a region conserved in higher plants. *EF1*α fragments were sequenced, and a forward primer and a reverse primer, spanning an intron, were designed on the sequences. The specificities of qPCR primers were confirmed by dissociation curve analysis and gel electrophoresis of PCR products. Primer information is summarized in Supplementary Table S1.

### Estimation of *IREG2* Copy Number

Genomic DNA was extracted from young leaves of plants grown in a soil culture, and then *IREG2* copy number was estimated by qPCR as described previously (Ueno et al., 2011; Milner et al., 2014). The qPCR protocol was as described in the section “Quantitative Reverse Transcription (RT)-PCR Assay,” except that the genomic DNA was used as the template instead of cDNA. Relative abundance of the *IREG2* gene was normalized with that of the *SHR* gene (AT4G37650), which is a putative single copy gene, or the expressed sequence tag–based indel marker RR11nr025, of *N. caerulescens* (Ueno et al., 2011; Milner et al., 2014). Primer sequences are summarized in Supplementary Table S1.

### Plant Transformation

The open-reading frames of *NcIREG2* and *NjIREG2* were amplified from cDNA by PCR with PrimeSTAR HS DNA Polymerase using a 5′ primer with *Kpn*1 site and 3′ primer with *Xho*1 site (Supplementary Table S1). PCR products were cloned into the pMD20-T vector by TA cloning (Takara Bio. Inc). The resulting *IREG2*-pMD-T plasmids were extracted from three independent colonies of *E. coli* and then sequenced. *IREG2* fragments were subcloned into pENTR4 (Invitrogen) at the *Kpn*1–*Xho*1 sites, and subsequently subcloned into pGWB2 plant overexpression vector carrying the CaMV 35S promoter (Nakagawa et al., 2007) using Gateway LR Clonase II Enzyme mix (Invitrogen). The plasmid constructs were transferred into *Agrobacterium tumefaciens* strain GV3101 (pMP90), and the obtained transformants were used to transform the *ireg2-1* homozygous line using the standard floral dip method. T1 transformants were selected on agar plates containing 10 μg mL^–1^ hygromycin, and then transferred to a soil culture. T2 progenies of four independent T1 lines were used for the following experiments. Fifteen seedlings of each T2 line grown on agar plates for 2 weeks (without hygromycin) were pooled and subjected to qRT-PCR. The relative expression level of *IREG2* was calculated using *EF1*α as the internal reference gene.

### Determination of Ni Tolerance and Accumulation in Transgenic Plants

Seeds were surface-sterilized and sown on agar plates supplemented with 0–70 μM NiCl_2_ (without hygromycin) and cultured for 14 days. After exposure, seedlings were pooled for each line and growth and Ni concentrations in roots and shoots were determined as described above.

### Yeast Transformation and Ni Tolerance Assay

The *IREG2*-pMD-T plasmids were digested with *Kpn*I, and the plasmid ends were blunted by T4 DNA polymerase. The resulting linear plasmids were digested with *Xho*I, and *IREG2* fragments were isolated by gel electrophoresis. The pKT10-Gal-HA-BS yeast expression vector was digested with *Eco*RI, and the plasmid ends were blunted by a Klenow fragment; the plasmid was then digested by *Sal*I. *IREG2* fragments were ligated to the blunt end and *Sal*I-digested end of pKT10-Gal-HA-BS. The obtained plasmids were transformed into *Saccharomyces cerevisiae* strain BY4741 (*MATa his3 leu2 met15 ura3*) by the standard method. The transformants were selected on a yeast nitrogen base (YNB) uracil minus medium, which consisted of 0.67% YNB, 2% glucose, and appropriate amino acids. The assay for Ni tolerance was performed as described by Nishida et al. (2011) using YNB uracil minus media containing 0 or 750 μM NiCl_2_, and 2% galactose (pH 5.5).

### Nicotianamine Analysis

Plants cultured hydroponically were exposed to 0 or 200 NiCl_2_ for a week. After exposure, roots were harvested and subjected to nicotianamine extraction following Nozoye et al. (2014). Nicotianamine was measured using high-performance liquid chromatography (HPLC) as described previously (Mori and Nishizawa, 1987).

## Results

### Difference in Ni Accumulation Between *N. japonica* and *N. caerulescens*

**FIGURE 1:**
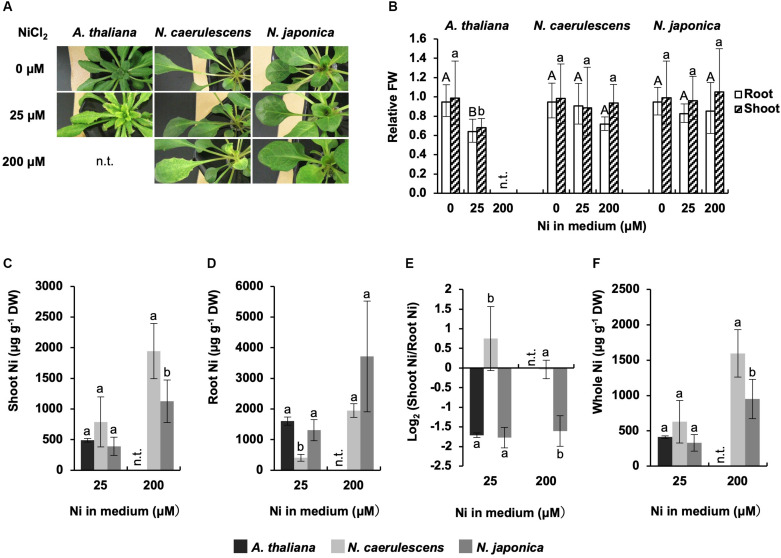
Ni tolerance and accumulation. Plants were exposed to the indicated concentrations of NiCl_2_ for 7 days in hydroponic culture. **(A)** Plants after Ni treatment. **(B)** Fresh weight (FW) relative to un-treated control. **(C,D)** Ni concentration expressed relative to dry weight (DW) in shoots **(C)** and roots **(D)**. **(E)** Ratio of shoot Ni to root Ni concentration. **(F)** Ni concentration in whole plants. Values are geometric **(B,E)** or arithmetic **(C,D,F)** means ± SD of four biological replicates. Different letters indicate a statistically significant difference (P < 0.05; ANOVA, Tukey–Kramer) between different Ni treatments in each part **(B)** or different species in each treatment **(C–F)**. n.t., not tested. Mean values of shoot FW/root FW under 0 μM Ni: 2.64 g/1.14 g (*A. thaliana*); 2. 02 g/1.01 g (*N. caerulescens*); 1.87 g/0.78 g (*N. japonica*).

*Noccaea japonica*, *N. caerulescens* (ecotype Ganges), and *A. thaliana* were exposed to 0, 25, or 200 μM Ni for a week in hydroponic culture, and Ni accumulation and symptoms were compared; *A. thaliana* was not tested at 200 μM Ni because this high concentration was lethal for this species. Following 25 μM Ni treatment, *A. thaliana* showed severe chlorosis, a typical symptom of Ni-induced Fe deficiency, over the whole shoot, and the growth in roots and shoots was significantly reduced compared with the un-treated controls (Figures 1A,B). In contrast, both *N. japonica* and *N. caerulescens* showed no visible symptoms or growth reduction, confirming that these *Noccaea* plant types have extremely high tolerance to Ni. Following 200 μM Ni treatment, *N. caerulescens* but not *N. japonica* exhibited chlorosis between the veins of young leaves; neither plant types showed significant growth reduction (Figures 1A,B and Supplementary Figure S1). Ni-induced Fe deficiency induces *IRT1* expression in roots and accelerates Fe absorption. This means that Fe concentration in roots increases with Ni concentration in the medium (Nishida et al., 2011, 2012). Here, Fe concentration in roots was significantly increased by treatment with 25 μM Ni in *N. caerulescens* (Supplementary Figure S1B) but not *N. japonica*. Taken together, the data for chlorosis symptoms and root Fe concentration indicate that *N. caerulescens* shows higher Ni sensitivity than *N. japonica*.

Ni concentration in shoots was significantly higher in *N. caerulescen*s than *N. japonica* following treatment with 200 μM Ni; the same tendency was observed for 25 μM Ni but the difference was not significant (Figure 1C). In contrast, Ni concentration in roots was significantly lower in *N. caerulescens* than *N. japonica* following treatment with either 25 or 200 μM Ni (Figure 1D). When the Ni levels in shoots and roots were compared, shoot Ni concentration was similar to or higher than root Ni concentration in *N. caerulescens* individuals, whereas shoot Ni concentration was lower than root Ni concentration in all *N. japonica* individuals tested. The ratio of shoot Ni to root Ni in *N. caerulescens* was significantly higher than that in *N. japonica* (Figure 1E). These results indicate that the activity of Ni translocation from roots to shoots was higher in *N. caerulescens* than in *N. japonica*. Following treatment with 200 μM Ni, the Ni concentration in whole plants was significantly higher in *N. caerulescens* than in *N. japonica* (Figure 1F) indicating that Ni absorption activity was higher in *N. caerulescens.*

**FIGURE 2:**
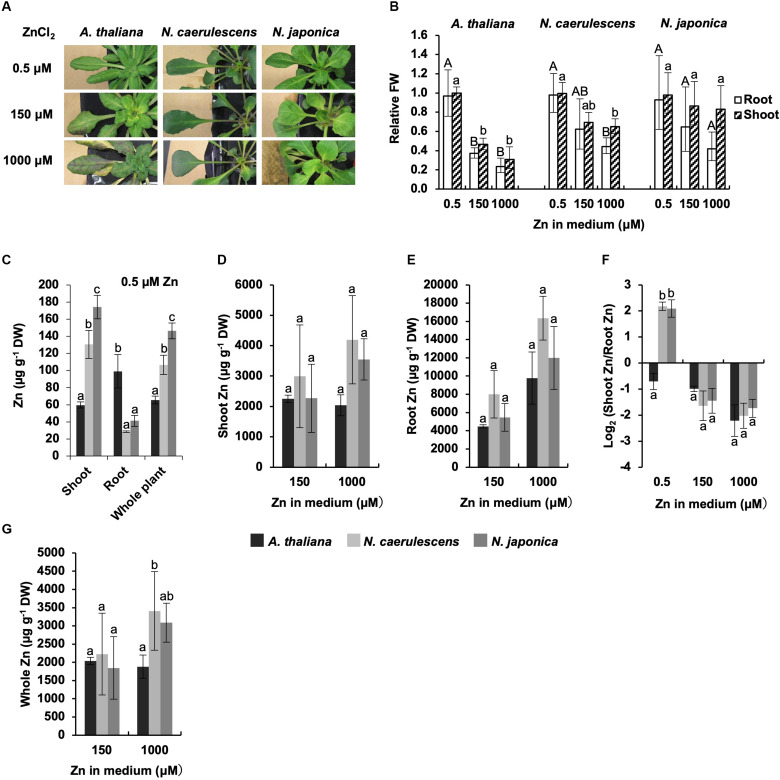
Zn tolerance and accumulation. Plants were exposed to the indicated concentrations of ZnCl_2_ for 7 days in hydroponic culture. **(A)** Plants after Zn treatments. **(B)** Fresh weight (FW) relative to un-treated control. **(C)** Zn concentration expressed relative to dry weight (DW) in shoots, roots, and whole plants under normal Zn condition (0.5 μM ZnCl_2_). **(D,E)** Zn concentrations in shoots **(D)** and root **(E)** under excess Zn conditions (150 and 1000 μM ZnCl_2_). **(F)** Ratio of shoot Zn to root Zn. **(G)** Zn concentration in whole plants. Values are geometric **(B,F)** or arithmetic **(C–E,G)** means ± SD of four biological replicates. Different letters indicate a statistically significant difference (P < 0.05; ANOVA, Tukey–Kramer) between different Zn treatments in each part **(B)**, different species in each part **(C)**, or different species in each treatment **(D–G)**. n.t., not tested. Mean values of shoot FW/root FW under 0.5 μM Zn: 3.04 g/1.08 g (*A. thaliana*); 2. 72 g/1.67 g (*N. caerulescens*); 1.36 g/0.67 g (*N. japonica*).

We also investigated Zn accumulation and symptoms in *N. japonica*, *N. caerulescens*, and *A. thaliana* following treatment with a normal Zn concentration(0.5 μM Zn) or excess Zn (150 or 1000 μM Zn). Growth of*A. thaliana* was greatly inhibited by 150 μMZn treatment compared with 0.5 μM Zn treatment, and serious necrosis developed in leaves following 1000 μM Zn treatment (Figure 2A). In contrast, both *N. japonica* and *N. caerulescens* showed high Zn tolerance, though there was a tendency for a slight decrease in growth under excess Zn conditions (Figures 2A,B and Supplementary Figure S2); this decrease was significant for 1000 μM Zn in *N. caerulescens* only. Shoot Zn concentrations in the *Noccaea* species were over twice that in *A. thaliana* under the normal Zn condition, while root Zn concentrations were lower in the *Noccaea* species than in *A. thaliana* (Figure 2C). These differences were statistically significant, confirming higher activity of root-to-shoot Zn translocation in the *Noccaea* species. In the comparison between *Noccaea* species, Zn concentrations in shoots and whole plants were significantly higher in *N. japonica* than in *N. caerulescens* under the normal Zn condition (Figure 2C), but no significant difference in Zn accumulation was observed under excess Zn conditions (Figures 2D,E,G). There was no difference in the ratio of shoot Zn to root Zn between *N. japonica* and *N. caerulescens* under excess Zn conditions (Figure 2F).

### Differences in *IREG2* Expression Level and Gene Copy Number Between *N. japonica* and *N. caerulescens*

The observed significant difference between *N. japonica* and *N. caerulescens* in terms of Ni, but not Zn, translocation from roots to shoots, suggests that the inter-species difference in Ni translocation is due to a Ni-specific mechanism. The efficiency of Ni translocation from roots to shoots depends on the abundances of vacuolar Ni^2+^ transporter IREG2 and metal chelator nicotianamine in roots. Nicotianamine accumulation in roots affects translocation of both Ni and Zn (Mari et al., 2006; Uraguchi et al., 2019), whereas IREG2 transports Ni^2+^ but not Zn^2+^. Therefore, we considered IREG2 to be the more likely candidate for the Ni-specific mechanism. We cloned the cDNAs of *IREG2* from the roots of *N. japonica* and *N. caerulescens* (termed *NjIREG2* and *NcIREG2*, respectively), and determined their sequences (Supplementary Figure S3). From *N. japonica*, three different *IREG2* sequences were obtained (Supplementary Figure S4). These sequences were almost identical (>99.5% identity), and therefore one of them was used for further analysis. A single *IREG2* sequence was obtained from *N. caerulescens*. The polypeptides encoded by *NjIREG2* and *NcIREG2* were almost identical to each other (99.4% identity) and consisted of 501 aa residues (Supplementary Figure S5). NjIREG2 and NcIREG2 shared considerable sequence identity (79%) with AtIREG2 (512 aa), suggesting that the functional role of IREG2 is conserved between the two *Noccaea* plant types.

**FIGURE 3:**
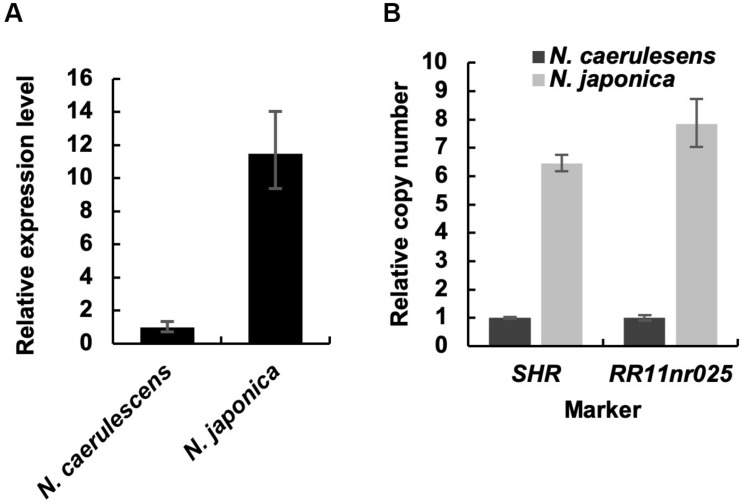
Root expression level and genomic copy number of *IREG2* in the two *Noccaea* plant types. **(A)** Plants were exposed to 200 μM NiCl_2_ for 7 days in hydroponic culture, and the roots were subjected to qRT-PCR. The relative expression levels of *IREG2* were calculated using *EF1α* as the internal reference gene. **(B)** The copy number of *IREG2* per genome was quantified by qPCR and normalized with the expressed sequence tag–based indel marker *RR11nr025* and the gene *SHR*. Values are means ± SD of three biological replicates.

We compared the expression level of *IREG2* in roots between *N. caerulescens* and *N. japonica* using qRT-PCR. The expression level in *N. japonica* was over 10-fold that in *N. caerulescens* following treatment with 200 μM Ni (Figure 3A). This suggests that the *IREG2* activity that sequesters Ni in root vacuoles is higher and Ni translocation to shoots is lower in *N. japonica* than in *N. caerulescens*. We also compared the genomic copy number of *IREG2* between the two *Noccaea* plant types. The abundances of *IREG2* gene relative to the abundance of the putative single copy gene *SHR* or the indel marker site RR11nr025 were compared between the two plant types. The copy number of *IREG2* per genome in *N. japonica* was estimated to be 6–8 times that in *N. caerulescens* (Figure 3B). This suggests that the higher expression of *IREG2* in *N. japonica* than in *N. caerulescens* is due to a higher copy number of *IREG2* in *N. japonica*.

### Confirmation of *NjIREG2* and *NcIREG2* Functions

**FIGURE 4:**
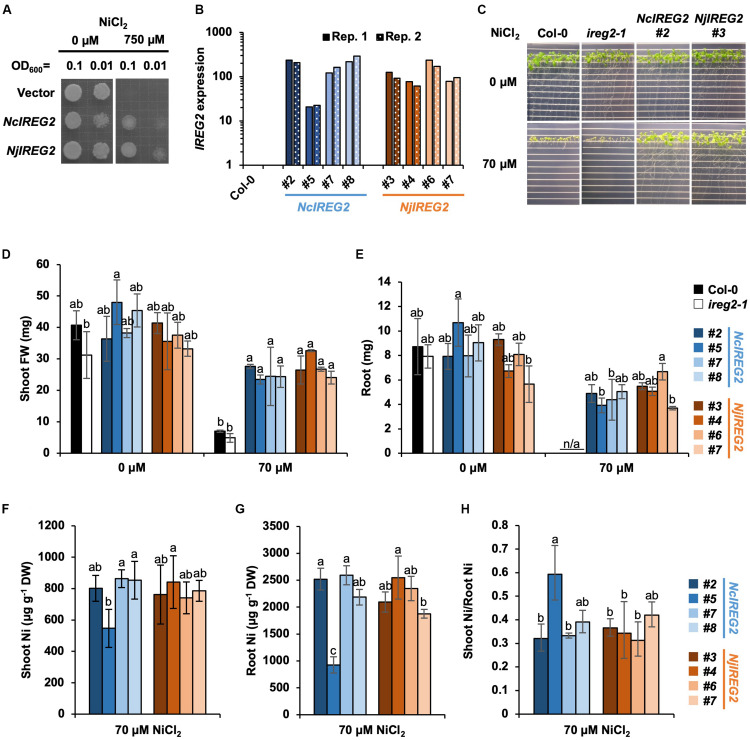
Heterologous expression of *NcIREG2* and *NjIREG2* in yeast and *A. thaliana*. **(A)** *Saccharomyces cerevisiae* cells were transformed with *NcIREG2*, *NjIREG2*, or empty vector and then grown on YNB media containing 0 or 750 μM NiCl_2_ at 30°C for 4 days. **(B)** Expression level of *IREG2* in the transgenic lines overexpressing *NcIREG2* or *NjIREG2*. The *ireg2-1* homozygous line was transformed with 35S:*NcIREG2* or 35S:*NjIREG2*, and four independent lines were selected for each construct. Relative expression levels of *IREG2* (Col-0 = 1) were determined by qRT-PCR. Data of each biological replicate (Rep. 1 and Rep. 2) are shown. **(C–E)** Growth of 14-day-old plants after the treatments. **(F,G)** Ni concentration expressed relative to dry weight (DW) in roots and shoots. **(H)** Ratio of shoot Ni to root Ni concentration. Values are means ± SD of four biological replicates. Different letters indicate a statistically significant difference (P < 0.05) among lines in each Ni treatment (ANOVA, Tukey–Kramer).

According to Schaaf et al. (2006), heterologous expression of *AtIREG2* increases the Ni tolerances of *S. cerevisiae*, and also overexpression of *AtIREG2* increases the Ni tolerance of *A. thaliana*. Therefore, to determine whether *NjIREG2* and *NcIREG2* are functional homologs of *AtIREG2*, we first established *S. cerevisiae* strains expressing *NjIREG2* or *NcIREG2* and assayed their Ni tolerance. Both strains showed increased Ni tolerance compared with the vector control strain (Figure 4A). We then evaluated the Ni tolerance of *A. thaliana* transgenic lines expressing *NcIREG2* or *NjIREG2*. cDNA fragments of *NjIREG2* and *NcIREG2* driven by the 35S promoter were separately introduced into the *ireg2-1* homozygous line, and four independent lines constitutively expressing *NcIREG2* or *NjIREG2* were selected (Figure 4B). The Col-0 and *ireg2-1* lines showed severe growth inhibition under 70 μM Ni compared with un-treated control, whereas the transgenic lines expressing *NjIREG2* or *NcIREG2* showed greatly increased Ni tolerance (Figures 4C–E). These results confirmed that *NjIREG2* and *NcIREG2* encode functional IREG2 proteins. Moreover, line *NcIREG2#5*, in which *IREG2* expression level was considerably lower than in the other *NcIREG2-* or *NjIREG2*-expressing lines (Figure 4B), showed significantly lower root Ni accumulation compared with the others (Figure 4G). In addition, the ratio of shoot Ni to root Ni in *NcIREG2#5* tended to be higher than that in the other lines (Figures 4F–H). These results indicate that the ability to sequester Ni in root vacuoles is lower in *NcIREG2#5* than in the other lines, and supports the notion that the expression level of *IREG2* determines the efficiency of root-to-shoot Ni translocation. Except for *NcIREG2#5*, there was no significant difference in Ni concentrations in roots and shoots between the *NjIREG2*-expressing lines and *NcIREG2*-expressing lines, suggesting that Ni sequestration activity is comparable between NcIREG2 and NjIREG2.

## Discussion

**FIGURE 5:**
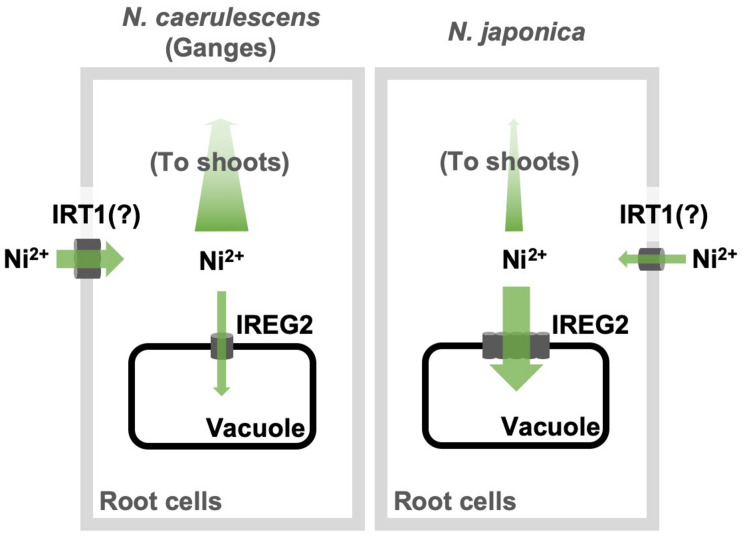
Schematic model of IREG2-dependent mechanism causing differential Ni accumulation in *N. japonica* and *N. caerulescens*. The gray rectangles and black rectangles denote the root cell membranes and the vacuole membranes, respectively.

Here, we showed that the efficiency of root-to-shoot Ni translocation is significantly lower in *N. japonica* than in *N. caerulescens* (ecotype Ganges), and that this is possibly due to higher *IREG2* expression in *N. japonica* than in *N. caerulescens*. Moreover, we showed that the genomic copy number of *IREG2* is much higher in *N. japonica* than in *N. caerulescens*, which likely causes the higher expression of *IREG2* in *N. japonica*. The multiple *IREG2* transcripts obtained from *N. japonica* (Supplementary Figure S4) could be derived from the multiplicated *IREG2*. These findings indicate that *IREG2* could be a genetic factor determining variation in shoot Ni hyperaccumulation. A hypothetical model of the IREG2-dependent mechanism causing differential shoot Ni accumulation in the two *Noccaea* plant types is shown in Figure 5: Ni is absorbed by IRT1 (or unknown Ni transporters) into root cells; a part of the Ni absorbed is sequestered in root vacuoles by IREG2, and the other part is loaded into the xylem and translocated from roots to shoots; in *N. japonica*, the elevated expression of *IREG2* in roots increases Ni accumulation in root vacuoles and decreases the amount of Ni to be loaded into xylem, thereby reducing the amount of Ni translocated from roots to shoots. A similar mechanism was shown for Cd accumulation in rice and *A. thaliana*: HMA3 suppresses Cd translocation from roots to shoots by sequestering Cd in the root vacuoles and determines variation in shoot Cd accumulation among cultivars or accessions (Ueno et al., 2010; Chao et al., 2012). Metal sequestration in root vacuoles is probably a major trait determining the efficiency of metal translocation from roots to shoots. We observed that the Ni concentration in the whole plant was higher in *N. caerulescens* than *N. japonica* following 200 μM Ni treatment (Figure 1F), indicating that Ni absorption activity was higher in *N. caerulescens*. Both the higher Ni absorption activity and the lower *IREG2* expression in *N. caerulescens* likely contribute to its higher sensitivity to Ni compared with *N. japonica* (Figure 1A and Supplementary Figure S1). *IRT1* expression is known to accelerate Ni absorption in *A. thaliana* (Nishida et al., 2012). We therefore hypothesize that more Ni is absorbed under excess Ni conditions in *N. caerulescens* than *N. japonica* because the induction of *IRT1* by Ni-induced Fe deficiency is higher in *N. caerulescens*. This hypothesis assumes that IRT1 is predominantly responsible for Ni absorption from soils. Further analyses are necessary to confirm the involvement of IRT1 in Ni hyperaccumulation in *N. caerulescens* and *N. japonica*.

In Ni hyperaccumulators, shoot Ni accumulation depends in part on nicotianamine accumulation in the roots: i.e., more nicotianamine accumulated in the roots increases the mobility of Ni in root cells and facilitates Ni translocation from roots to shoots (Curie et al., 2008). However, although the ratio of shoot Ni to root Ni concentration (an indicator of root-to-shoot translocation) was higher in *N. caerulescens* than *N. japonica*, we observed no significant difference in the ratio of shoot Zn to root Zn concentration between the two *Noccaea* plant types. If root nicotianamine were responsible for this difference, one might expect the root nicotianamine concentration to be higher in *N. caerulescens* than *N. japonica*, but this was not the case (Supplementary Figure S6). This finding provides further evidence that nicotianamine, which affects both Ni and Zn translocation, is not responsible for the difference in root-to-shoot Ni translocation between the two plant types. Richau et al. (2009) reported that shoot Ni accumulation in a Ni-hyperaccumulating *N. caerulescens* accession depends on the enhanced accumulation of free histidine in the roots: i.e., chelation of Ni by free histidine accumulated in the roots inhibits Ni sequestration into the root vacuoles and promotes Ni translocation from roots to shoots. However, Persans et al. (1999) denied the implication of root histidine accumulation in shoot Ni hyperaccumulation in the Ni hyperaccumulator *N. goesingenese*. Although the role of free histidine in Ni hyperaccumulation is still controversial, the possibility that free histidine is another factor determining variation in Ni hyperaccumulation in *Noccaea* cannot be ruled out.

It has been reported that *N. caerulescens* shows more efficient root-to-shoot Ni translocation compared with metal non-accumulator plants such as *A. thaliana* (Assunção et al., 2003; Mari et al., 2006). Here, we showed that the ratio of shoot Ni to root Ni concentration was significantly higher in *N. caerulescens* than in *A. thaliana* (Figure 1), confirming a higher Ni translocation in *N. caerulescens*. This difference is probably due to elevated nicotianamine accumulation in the roots of *N. caerulescens* as reported previously (Mari et al., 2006). A transcriptome analysis showing that *IREG2* is not differentially expressed between *N. caerulescens* (a Zn/Pb-mine ecotype) and *A. thaliana* Col-0 (van de Mortel et al., 2006) suggests that *IREG2* expression is not involved in the difference in Ni translocation. In contrast, our data suggest that the efficiency of Ni translocation from roots to shoots in *N. japonica* is similar to that of *A. thaliana* following treatment with 25 μM Ni (Figure 1E). Root nicotianamine accumulation in *N. japonica* was comparable with that in *N. caerulescens*, but the potential for efficient Ni translocation caused by elevated nicotianamine accumulation in roots might be offset by the elevated *IREG2* expression. Efficient translocation of metals from roots to shoots is generally found in metal hyperaccumulators. However, our results imply that efficient Ni translocation is not necessary for Ni hyperaccumulation. Shoot Ni concentrations could reach the hyperaccumulation level in *N. japonica* growing in serpentine soils for a long time (Mizuno et al., 2003).

The expression levels of metal transporter genes (e.g., *HMA3*, *HMA4*, and *NRAMP1*) involved in shoot metal hyperaccumulation are elevated by gene duplication in metal hyperaccumulator species, and gene duplication is recognized as a mechanism facilitating evolution of metal hyperaccumulators (Hanikenne et al., 2008; Ueno et al., 2011; Milner et al., 2014). *IREG2* is multiplicated in *N. japonica* and accordingly *IREG2* expression is elevated. But, the elevated *IREG2* reduces root-to-shoot Ni translocation by sequestering Ni in the root vacuoles (Schaaf et al., 2006; Morrissey et al., 2009) and so does not contribute to the trait of efficient Ni translocation from roots to shoots. *N. japonica* grows in serpentine soil areas, whereas *N. caerulescens* Ganges, the ecotype studied here, grows in Zn/Pb mine soil areas. *IREG2* expression in *N. caerulescens* is higher in an ecotype from serpentine soils than in ecotypes from Zn/Pb mine soils or non-metalliferous soil (Halimaa et al., 2014). We assume that elevation of *IREG2* expression was required to survive under excess Ni conditions. Whole genome sequence analysis of natural populations of the non-accumulator *Arabidopsis lyrata* revealed that a non-synonymous substitution in *IREG2* is strongly linked to adaptation to serpentine soils (Turner et al., 2010). *IREG2* might be a major target to be duplicated or modified for adaptation to Ni-rich soils in wild plants. For future study, it would be important to investigate the involvement of *IREG2* in intra- and inter-specific variations in Ni hyperaccumulation pattern and Ni tolerance using a wide variety of species and accessions of *Brassicaceae* metal hyperaccumulators.

## Data Availability Statement

The raw data supporting the conclusions of this article will be made available by the authors, without undue reservation, to any qualified researcher.

## Author Contributions

SN designed the study and wrote the manuscript. SN, RT, SI, JY, and HN contributed to analyses. HN and NF assisted in the preparation of the manuscript. All authors contributed to data interpretation and approved the final version of the manuscript.

## Conflict of Interest

The authors declare that the research was conducted in the absence of any commercial or financial relationships that could be construed as a potential conflict of interest. The handling Editor declared past co-authorship, with one of the authors, HN.
