# Supplementary material for: Elevated Expression of Vacuolar Nickel Transporter Gene IREG2 Is Associated With Reduced Root-to-Shoot Nickel Translocation in Noccaea japonica
Source: Front Plant Sci. 2020 Jun 3;11:610. doi: 10.3389/fpls.2020.00610 (PMC7283525; doi:10.3389/fpls.2020.00610)
Supplement: Supplementary file 1 [file Data_Sheet_1.PDF]

A

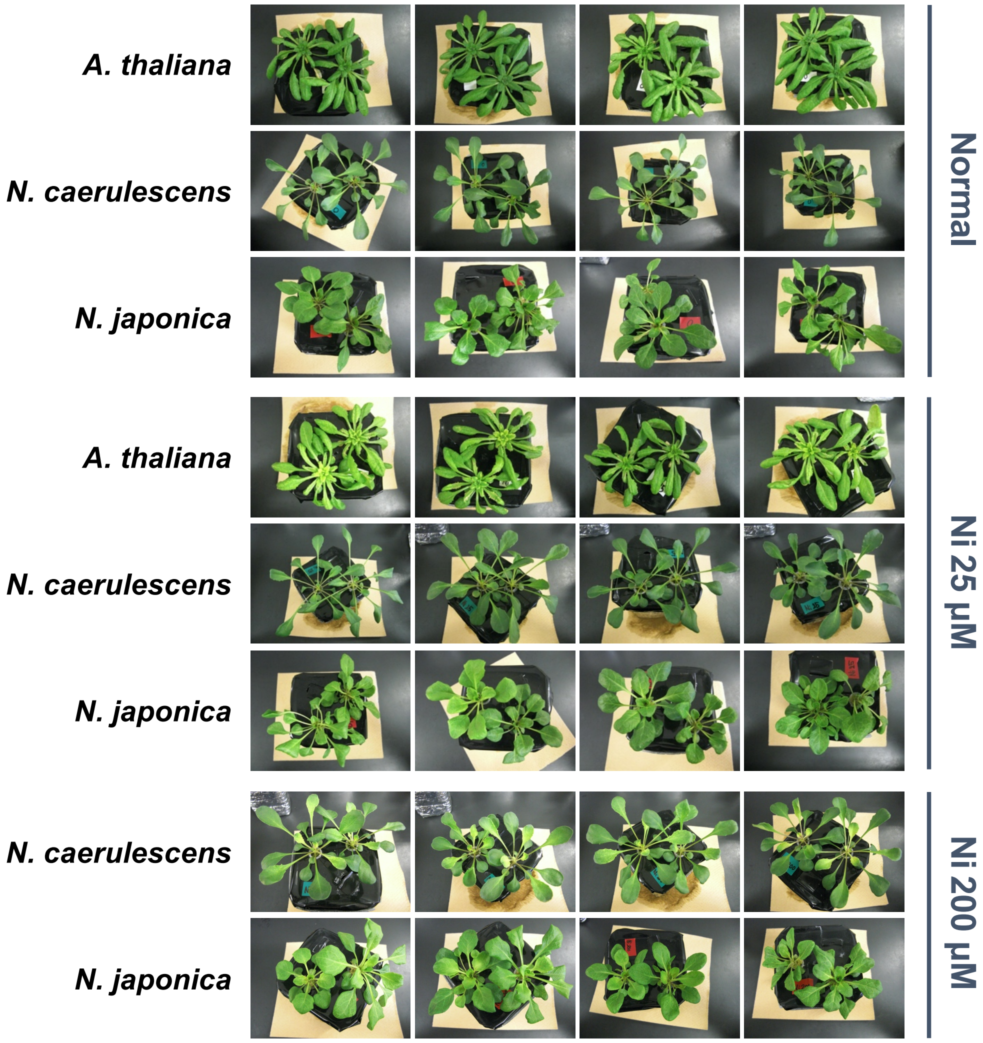

B

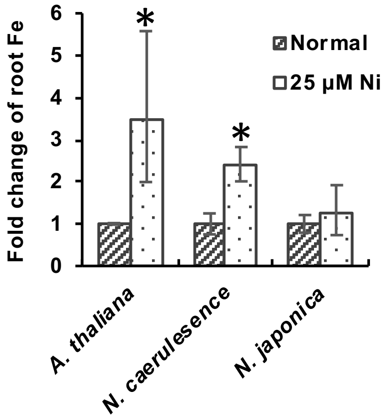

Figure S1 Ni tolerance of *Arabidopsis thaliana*, *Noccaea caerulescens* and *Noccaea japonica*. (A) Individual plants after treatment with 0 µM Ni (normal condition) or 25 or 200 µM Ni (N = 4, each group). (B) Fold change of Fe concentration in roots (25 µM Ni condition/normal condition). Values are geometric means ± SD of four biological replicates. Asterisks indicate a statistically significant difference ($P < 0.05$) between the normal condition and 25 µM Ni (Student's t-test).

A

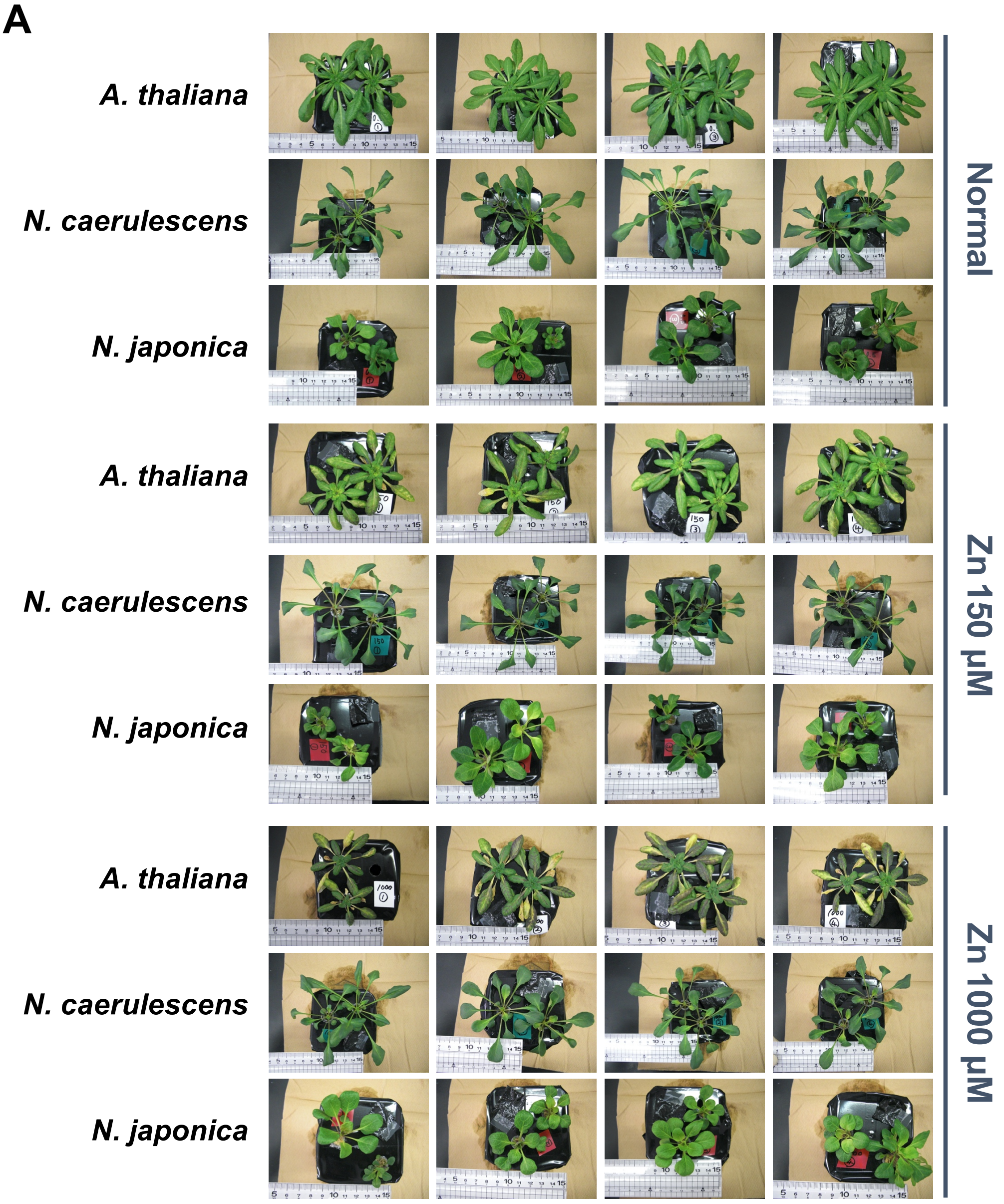

B

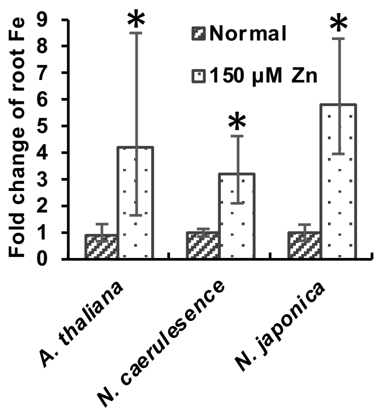

Figure S2 Zn tolerance of *A. thaliana*, *N. caerulescens*, and *N. japonica*.
(A) Individual plants after treatment with 0.5 μM Zn (normal condition) or 150 or 1000 μM Zn (N = 4, each group). (B) Fold change of Fe concentration in roots (150 μM Zn condition/normal condition). Values are geometric means ± SD of four biological replicates. Asterisks indicate a statistically significant difference ($P < 0.05$) between the normal condition and 150 μM Zn (Student's t-test).

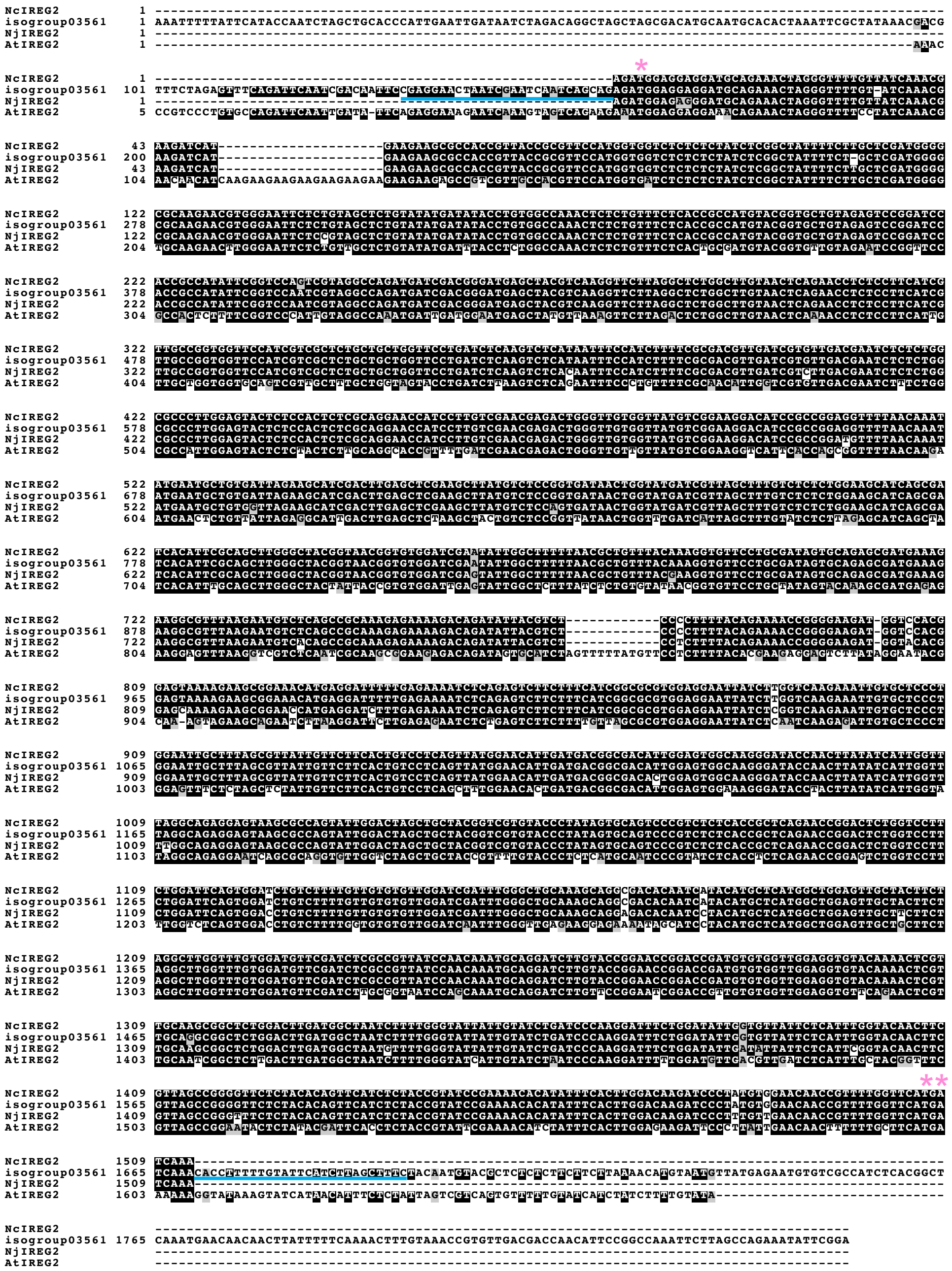

Figure S3 Alignment of *IREG2* cDNA sequences.

The sequences of *IREG2* cDNAs from the roots of *N. japonica* and *N. caerulescens* (termed *NjIREG2* and *NcIREG2*, respectively) and from *A. thaliana* (*AtIREG2*; AT5G03570), and the *IREG2* sequence Nc_isotig06993_isogroup03561, which was assembled by *de novo* RNA sequencing analysis of *N. caerulescens* (Lin et al. 2014; BioProject: PRJNA237055), were aligned by using Clustal W. Start (*) and stop (**) codons are indicated. Identical and similar nucleotides are shaded in black and gray, respectively. Primer sites in Nc_isotig06993_isogroup03561 used for cloning *NcIREG2* and *NjIREG2* are underlined.

A

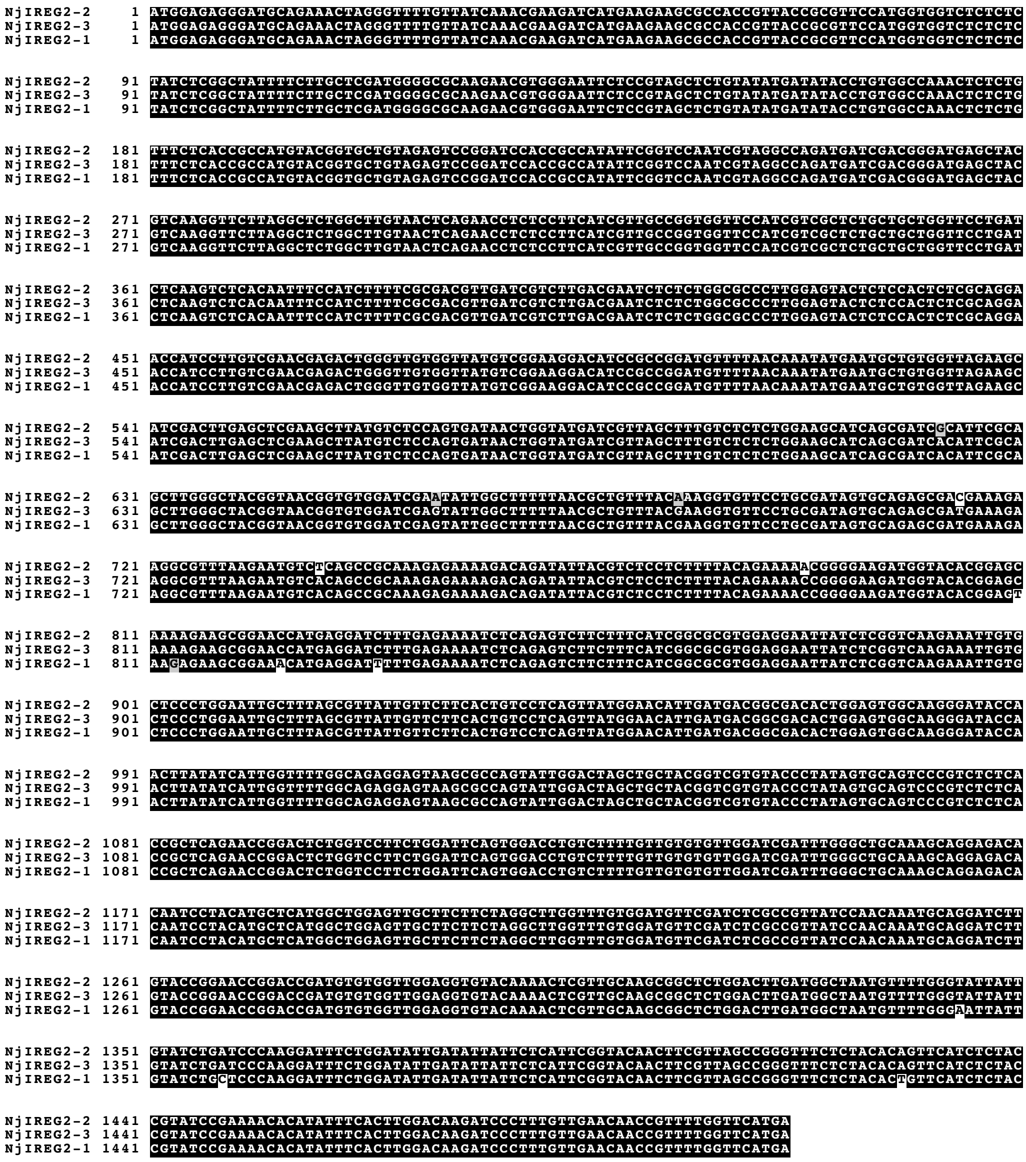

Figure S4 Multiple sequences of *NjIREG2* cDNAs.
(A) The sequences of *NjIREG2* cDNAs were aligned by using Clustal W. Identical and similar residues are shaded in black and gray, respectively. *NjIREG2-3* was used for further analysis. (B) The alignment of amino acid sequences encoded by NjIREG2 cDNAs. *NjIREG2-3* was used for further analysis.

B

```
NjIREG2-1     1 MERDAETRVLLSNEDHEEAPPLPRSMVVSLYLGYFLARWGARTWEFSVALYMIYLWPNSL
NjIREG2-3     1 MERDAETRVLLSNEDHEEAPPLPRSMVVSLYLGYFLARWGARTWEFSVALYMIYLWPNSL
NjIREG2-2     1 MERDAETRVLLSNEDHEEAPPLPRSMVVSLYLGYFLARWGARTWEFSVALYMIYLWPNSL

NjIREG2-1    61 FLTAMYGAVESGSTAIFGPIVGQMIDGMSYVKVLRLWLVTQNLSFIVAGGSIVALLLVPD
NjIREG2-3    61 FLTAMYGAVESGSTAIFGPIVGQMIDGMSYVKVLRLWLVTQNLSFIVAGGSIVALLLVPD
NjIREG2-2    61 FLTAMYGAVESGSTAIFGPIVGQMIDGMSYVKVLRLWLVTQNLSFIVAGGSIVALLLVPD

NjIREG2-1   121 LKSHNFHLFATLIVLTNLSGALGVLSTLAGTILVERDWVVVMSEGHPPDVLTNMNAVVRS
NjIREG2-3   121 LKSHNFHLFATLIVLTNLSGALGVLSTLAGTILVERDWVVVMSEGHPPDVLTNMNAVVRS
NjIREG2-2   121 LKSHNFHLFATLIVLTNLSGALGVLSTLAGTILVERDWVVVMSEGHPPDVLTNMNAVVRS

NjIREG2-1   181 IDLSSKLMSPVITGMIVSFVSLEASAITFAAWATVTVWIEYWLFNAVYEGVPAIVQSDER
NjIREG2-3   181 IDLSSKLMSPVITGMIVSFVSLEASAITFAAWATVTVWIEYWLFNAVYEGVPAIVQSDER
NjIREG2-2   181 IDLSSKLMSPVITGMIVSFVSLEASAIAFAAWATVTVWIEYWLFNAVYKGVPAIVQSDER

NjIREG2-1   241 RRLRMSQPQREKTDITSPLLQKTGEDGTRSKRSGNMRIFEKISESSFIGAWRNYLGQEIV
NjIREG2-3   241 RRLRMSQPQREKTDITSPLLQKTGEDGTRSKRSGTMRIFEKISESSFIGAWRNYLGQEIV
NjIREG2-2   241 RRLRMSQPQREKTDITSPLLQKNGEDGTRSKRSGTMRIFEKISESSFIGAWRNYLGQEIV

NjIREG2-1   301 LPGIALALLFFTVLSYGTLMTATLEWQGIPTYIIGFGRGVSASIGLAATVVYPIVQSRLS
NjIREG2-3   301 LPGIALALLFFTVLSYGTLMTATLEWQGIPTYIIGFGRGVSASIGLAATVVYPIVQSRLS
NjIREG2-2   301 LPGIALALLFFTVLSYGTLMTATLEWQGIPTYIIGFGRGVSASIGLAATVVYPIVQSRLS

NjIREG2-1   361 PLRTGLWSFWIQWTCLLLCVGSIWAAKQETQSYMLMAGVASSRLGLWMFDLAVIQQMQDL
NjIREG2-3   361 PLRTGLWSFWIQWTCLLLCVGSIWAAKQETQSYMLMAGVASSRLGLWMFDLAVIQQMQDL
NjIREG2-2   361 PLRTGLWSFWIQWTCLLLCVGSIWAAKQETQSYMLMAGVASSRLGLWMFDLAVIQQMQDL

NjIREG2-1   421 VPEPDRCVVGGVQNSLQAALDLMANVLGIIVSAPKDFWILILFSFGTTSLAGFLYTVHLY
NjIREG2-3   421 VPEPDRCVVGGVQNSLQAALDLMANVLGIIVSDPKDFWILILFSFGTTSLAGFLYTVHLY
NjIREG2-2   421 VPEPDRCVVGGVQNSLQAALDLMANVLGIIVSDPKDFWILILFSFGTTSLAGFLYTVHLY

NjIREG2-1   481 RIRKHIFHLDKIPLLNNRFGS
NjIREG2-3   481 RIRKHIFHLDKIPLLNNRFGS
NjIREG2-2   481 RIRKHIFHLDKIPLLNNRFGS
```

Figure S4 Multiple sequences of *NjIREG2* cDNAs.
(Continued.)

```
NcIREG2    1 MERDAETRVLLSNE-------DHEEAPPLPRSMVVSLYLGYFLARWGARTWEFSVALYMI
NjIREG2    1 MERDAETRVLLSNE-------DHEEAPPLPRSMVVSLYLGYFLARWGARTWEFSVALYMI
AtIREG2    1 MEEETETRVFLSNEQHQEEEEEEEEEPSLPRSMVISLYLGYFLARWGARTWEFSVALYMI

NcIREG2   54 YLWPNSLFLTAMYGAVESGSTAIFGPIVGQMIDGMSYVKVLRLWLVTQNLSFIVAGGSIV
NjIREG2   54 YLWPNSLFLTAMYGAVESGSTAIFGPIVGQMIDGMSYVKVLRLWLVTQNLSFIVAGGSIV
AtIREG2   61 YLWPNSLFLTAMYGVVESGSATLFGPIVGQMIDGMSYVKVLRLWLVTQNLSFIVAGGAVV

NcIREG2  114 ALLLVPDLKSHNFHLFATLIVLTNLSGALGVLSTLAGTILVERDWVVVMSEGHPPDVLTN
NjIREG2  114 ALLLVPDLKSHNFHLFATLIVLTNLSGALGVLSTLAGTILVERDWVVVMSEGHPPDVLTN
AtIREG2  121 ALLVVPDLKSQNFPVFATLVVLTNLSGAIGVLSTLAGTVLIERDWVVVMSEGHSPAVLTR

NcIREG2  174 MNAVVRSIDLSSKLMSPVITGMIVSFVSLEASAIAFAAWATVTVWIEYWLFNAVYKGVPA
NjIREG2  174 MNAVVRSIDLSSKLMSPVITGMIVSFVSLEASAITFAAWATVTVWIEYWLFNAVYEGVPA
AtIREG2  181 MNSVIRGIDLSSKLLSPVITGLIISFVSLRASAITFAAWATITVWIEYWLFISVYNGVPA

NcIREG2  234 IVQSDERRRLRMSQPQREKTDITS----PLLQKNGEDGTRSKRSGTMRIFEKISESSFIG
NjIREG2  234 IVQSDERRRLRMSQPQREKTDITS----PLLQKTGEDGTRSKRSGTMRIFEKISESSFIG
AtIREG2  241 IVQSDERRSLRSSQSQAEETDSASSFYVPLLHEEESYRNTQSRSRILRILERISESSFVS

NcIREG2  290 AWRNYLGQEIVLPGIALALLFFTVLSYGTLMTATLEWQGIPTYIIGFGRGVSASIGLAAT
NjIREG2  290 AWRNYLGQEIVLPGIALALLFFTVLSYGTLMTATLEWQGIPTYIIGFGRGVSASIGLAAT
AtIREG2  301 AWRNYLNQEIVLPGVSLALLFFTVLSFGTLMTATLEWKGIPTYIIGIGRGISAGVGLAAT

NcIREG2  350 VVYPIVQSRLSPLRTGLWSFWIQWTCLLLCVGSIWAAKQETQSYMLMAGVASSRLGLWMF
NjIREG2  350 VVYPIVQSRLSPLRTGLWSFWIQWTCLLLCVGSIWAAKQETQSYMLMAGVASSRLGLWMF
AtIREG2  361 VLYPLMQSRISPLRTGVWSFWSQWTCLLVCVGSIWVEKEKIASYMLMAGVAASRLGLWMF

NcIREG2  410 DLAVIQQMQDLVPEPDRCVVGGVQNSLQAALDLMANVLGIIVSDPKDFWILILFSFGTTS
NjIREG2  410 DLAVIQQMQDLVPEPDRCVVGGVQNSLQAALDLMANVLGIIVSDPKDFWILILFSFGTTS
AtIREG2  421 DLAVIQQMQDLVPESDRCVVGGVQNSLQSALDLMANLLGIIVSNPKDFWMLTLISFATVS

NcIREG2  470 LAGFLYTVHLYRIRKHIFHLDKIPLLNNRFGS
NjIREG2  470 LAGFLYTVHLYRIRKHIFHLDKIPLLNNRFGS
AtIREG2  481 LAGILYTIHLYRIRKHLFHLEKIPLLNNFFAS
```

Figure S5 Alignment of IREG2 protein sequences.
Amino acid sequences of NcIREG2, NjIREG2, and AtIREG2 were aligned by using Clustal W. Identical and similar residues are shaded in black and gray, respectively. Amino acid identity between NcIREG2 and NjIREG2 was 99.4% (3 aa difference), and that between AtIREG2 and both NcIREG2 and NjIREG2 was 79%.

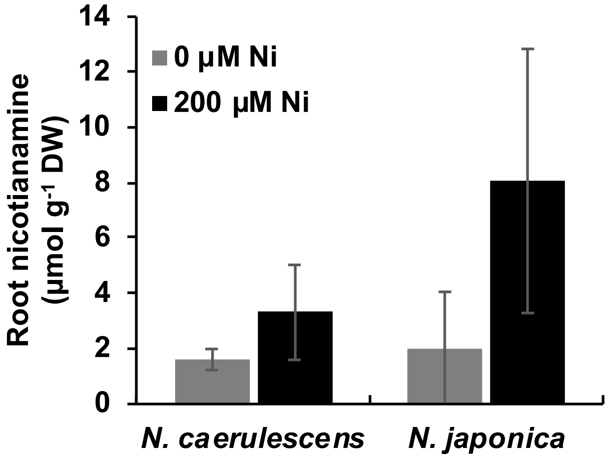

Figure S6 Nicotianamine accumulation in roots.
Plants were grown in hydroponic culture supplemented with 0 or 200 µM $NiCl_2$ for 1 week. Nicotianamine concentration in roots was determined by HPLC. Values are means ± SD of three biological replicates. There is no significant difference between plant species in each treatment condition (Student's t-test).
